# A New Flavonoid C-Glycoside from *Celtis australis* L. and *Celtis occidentalis* L. Leaves and Potential Antioxidant and Cytotoxic Activities

**DOI:** 10.3797/scipharm.1108-19

**Published:** 2011-10-06

**Authors:** Taha S. El-Alfy, Hamida M. A. El-Gohary, Nadia M. Sokkar, Mohammed Hosny, Dalia A. Al-Mahdy

**Affiliations:** 1 Department of Pharmacognosy, Faculty of Pharmacy, Cairo University, Kasr-El-Ainy, 11562, Cairo, Egypt; 2 Department of Pharmacognosy, Faculty of Pharmacy, Al-Azhar University, 11787, Cairo, Egypt

**Keywords:** *Celtis australis* L., *Celtis occidentalis* L., Flavonoid C-glycosides, DPPH, Xanthine oxidase, Lipid peroxidation, Cytotoxic activities

## Abstract

A major development over the past two decades has been the realization that free radical induced lipid peroxidation and DNA damage are associated with major health problems, e.g. cancer and ageing. Plant-derived antioxidants are increasingly found beneficial in protecting against these diseases. *Celtis australis* L. and *Celtis occidentalis* L. are two plants that have a variety of uses in folk medicine but have not been evaluated before for their antioxidant and cytotoxic properties. Therefore, the extracts of both plants’ leaves were investigated for these activities, as well as isolation of the bioactive compounds responsible for the activities. Molecular structures of the compounds were elucidated by UV, HRESIMS, 1D (^1^H and ^13^C) and 2D (^1^H-^13^C HSQC and ^1^H-^13^C HMBC) NMR analyses. The ethanolic and aqueous extracts, *n*-butanol fractions and the isolated major compound were tested for their antioxidant activity using DPPH radical scavenging assay, xanthine oxidase-induced generation of superoxide radical and lipid peroxidation assay by thiobarbituric acid-reactive substances (TBARS) method using rat tissue homogenates. Cytotoxic activities were studied using standard MTT assay. A novel flavonoid C-triglycoside, 4‴-α-rhamnopyranosyl-2″*-O-*β-d-galactopyranosylvitexin, was isolated from both plants’ leaves, together with seven known flavonoids. The *n*-butanol fractions and the major compound 2″*-O-*β-galactopyranosylvitexin showed significant antioxidant activities, more pronounced than the tested standards BHT and dl-α-tocopherol in most tests. All extracts showed variable cytotoxic activities. This study provides strong evidence for the antioxidant and cytotoxic activities of the extracts of *Celtis australis* L. and *Celtis occidentalis* L. leaves, which were attributed to the polar *n*-butanol fractions and the major isolated flavonoid 2″-galactosylvitexin.

## Introduction

*Celtis australis* L., Family Ulmaceae (Mediterranean hackberry), is a deciduous tree native to the Mediterranean region and southwestern Asia [1]. Decoctions of the leaves and fruits were used to astringe the mucous membrane in peptic ulcers, diarrhoea, and dysentery and as a remedy for heavy menstrual bleeding and colic [2, 3]. In Indian traditional medicine, the plant is an important remedy for bone fracture, contusions, sprains and joint pains [4].

*Celtis occidentalis* L., Family Ulmaceae (Hackberry, American Hackberry), is a medium-size deciduous tree native to North America. Native Americans used decoctions prepared from the bark as an aid in menses and to relieve sore throat and used the wood extract in treating jaundice [5, 6].

Previous investigations reported the isolation of acacetin 7*-O-*glucoside, isovitexin, cytisoside, 2″-α-rhamnopyranosylvitexin and 2″-α-rhamnopyranosyl-7*-O-*methylvitexin from the leaves of *Celtis australis* L. [7, 8], a sulphonated phenolic compound (celtisanin), apigenin, quercetin and quercetin-glucoside from the fruits [9] and a bacteriohopanoid from the barks [10]. Nothing was reported concerning *Celtis occidentalis* L.

Antioxidant and cytotoxic activities were reported before with other *Celtis species* e.g. *Celtis philippinensis* Blanco [11], *Celtis africana* Burm. f. [12], and *Celtis iguanae* (Jacq.) Sarg. [13]. According to the available literature, these activities were not investigated before in *Celtis australis* L. and *Celtis occidentalis* L. leaves.

Known isolated flavonoid C-glycosides e.g. vitexin, isovitexin, orientin and isoorientin were investigated before for their antioxidant activities [14–16].

Therefore, this study reports the isolation of a new flavonoid C-glycoside 8-(4‴-*α*-rhamnosyl-2″*-O-*β-d-galactopyranosylvitexin) along with seven known flavonoid glycosides (**1–7**). The antioxidant properties of the ethanolic and aqueous extracts were investigated since most of the herbal products available in the market are found in the form of tea bags or crude ethanolic extracts. Also the flavonoid-rich *n*-butanol fractions of the leaves as well as the major isolated flavonoid 6, were investigated for their antioxidant properties using 1,1-diphenyl-2-picrylhydrazyl (DPPH) radical scavenging assay, xanthine oxidase-induced generation of superoxide radical assay and measurement of FeSO_4_/H_2_O_2_-stimulated lipid peroxidation in rat tissue homogenates. The cytotoxic activity against different human cell lines was also studied.

## Results and Discussion

### Phytochemical investigation

Compound 8 was obtained as a yellow powder. The positive ESI-MS spectrum gave a molecular ion at m/z 741 (M + H)^+^, whereas the negative ESI-MS (M – H) – ion at m/z 739. These data together with the ^13^C NMR spectrum showing 33 signals indicated the molecular formula to be C_33_H_40_O_19_. The UV spectrum showed characteristic flavone absorption at 272 and 330 nm [17]. The ^1^HNMR spectrum indicated the presence of an apigenin skeleton substituted at C-8 through C-C linkage confirmed by the appearance of H-6 at δ 6.21. The appearance of three anomeric proton signals suggested a triglycoside moiety. The first anomeric proton at δ 4.76, J=9.9 Hz was assigned to a β-d-gluco-pyranose, the second anomeric proton at δ 3.99, J=9 Hz was assigned to a β-d-galacto-pyranose sugar while an α-l-rhamnopyranose moiety was represented by an anomeric proton at δ 4.90 and a doublet at δ 1.19, J=6 Hz assigned for CH_3_-6″″ of the rhamnopyranose.

The positive-ion MS/MS of compound **8** showed fragment ion peak at m/z 594.3 (M+H – 146)^+^ assignable to the loss of a desoxyhexose moiety from the parent ion which was suggestive of a linear triglycoside moiety. The desoxyhexose identified as rhamnose was found to be attached at position C-4‴ of the galactose sugar evidenced by its downfield shifting to δ 67.2 together with the upfield shifting of the anomeric proton to δ 101.4. HBMC spectrum was used to identify the carbon and proton signals. Correlations appearing between H-1″/C-8 confirmed attachment of the sugar unit through C-linkage to C-8 of the apigenin moiety. Also, correlations between 2″-H/C-1‴ and 1‴-H/C-2″ confirmed the linkage of the galactose to C-2″ of glucose. All these data were consistent with the structure apigenin-8-C-(4‴*-O-*rhamnosyl-2″-galactosyl)-glucopyranoside, (4‴-α-l-rhamnosyl-2″*-O-β*-d-galactopyranosylvitexin). To the best of our knowledge, this is the first report for the isolation of this compound in nature.

Compounds **1** and **2** were identified as vitexin and orientin [18]; compounds **3** and **4** were identified as isovitexin and isoorientin [18–20]; compound **5** was identified as rutin [17, 21]. ESIMS and NMR analyses (1D [^1^H and ^13^C] and 2D [^1^H-^13^C HSQC and ^1^H-^13^C HMBC]) identified compound **6** as 2″*-O-*β-galactopyranosylvitexin and compound **7** as 2″*-O-*β-d-galactopyranosylorientin. Their data were compared with that reported in literature [22, 23]. Compounds (**1–3** and **5–7**) were isolated for the first time from genus *Celtis*. Both studied *Celtis* species showed a similar flavonoidal pattern differing only in the amount present.

### In-vitro biological evaluation

#### DPPH radical scavenging activity [24]

All the tested samples had significant scavenging effects on the DPPH radical. The results recorded in table 2 indicated that 2″*-O-β*-galactopyranosylvitexin showed the highest activity among all the tested samples (84.8%), followed by the *n*-butanol fraction (70.3%) and the ethanolic extract (67.2%), and they were higher than α-tocopherol and BHT (66.5 and 55.3%, respectively). The results obtained for the *n*-butanol fractions and 2″-galactosylvitexin indicated that the flavonoidal content (C-flavonoid glycosides) is responsible for the scavenging effects, and this could be attributed to their hydrogen donating activity. In general, the phenolic OH is considered a scavenger of free radicals, and it consequently exhibits antioxidative activity [25].

#### Xanthine oxidase-induced generation of superoxide radical [26]

The tested samples inhibited superoxide-induced reduction of nitroblue tetrazolium, which depends on direct inhibition of xanthine oxidase enzyme as shown in table 2. The major isolated flavonoid, 2″*-O-β*-galactopyranosylvitexin, showed the highest activity with IC_50_=24.2 μM, followed by the *n*-butanol fraction of both plants with IC_50_=27.2 μM and 38.5 μM, respectively.

Phenolic compounds are known to inhibit generation of the superoxide anion radical (O^2•−^) in the hypoxanthine-xanthine oxidase system. The radical scavenging action of these phenolic compounds is through the formation of stable free radicals, which contribute to the inhibitory effects on lipid peroxidation and participate in the inhibition of (O^2•−^) generation. Since the *n*-butanol fractions is rich in apigenin and luteolin derivatives e.g. isovitexin, orientin and isoorientin which were reported to have activities regarding inhibition of xanthine oxidase enzyme ***[16, 27]. T***herefore, these fractions could be effective as natural antioxidants, through their double ability to inhibit xanthine oxidase activity and superoxide anion production, and could be considered an effective strategy in the treatment of inflammation.

#### FeSO_4_/H_2_O_2_-stimulated lipid peroxidation in rat tissue homogenate [28, 29]

For rat tissue homogenate (brain, heart and liver), the unstimulated control experiments i.e. the amount of thiobarbituric reactive substance (TBARS) formed in rat tissue homogenate (brain, heart and liver) were (0.44 ± 0.05, 0.25 ± 0.03 and 0.19 ± 0.02 nmol malondialdehyde, MDA/mg protein, respectively). After induction with FeSO_4_/H_2_O_2_, the amount of TBARS increased to 0.90 ± 2.55, 0.60 ± 2.16 and 0.52 ± 1.25 nmol malondialdehyde, MDA/mg protein, for brain, heart and liver, respectively. The tested samples significantly reduced malondialdehyde (MDA) formation in the presence of FeSO_4_-H_2_O_2_ in tissue homogenates indicating anti-lipid peroxidation activities. The inhibition percentages were in the range of (55.10–80.85%), (52.40–71.50%) and (43.90–78.25%) in brain, heart and liver rat tissue homogenates, respectively as recorded in table 3.

It was interesting to note that the inhibition effects produced by the tested samples were more pronounced for brain tissue homogenates than liver and heart tissue homogenates, which could be especially beneficial in treatment of Alzheimer’s disease in cases with oxidative stress due to elevated levels of TBARS [30]. The ethanolic extract and 2″-*O*-β-galactopyranosylvitexin showed the highest inhibition activity against FeSO_4_/H_2_O_2_-stimulated lipid peroxidation in brain rat tissue homogenate (80.85 and 80.78%), respectively, which was higher than both reference standards. Since dl-α-tocopherol is thought to be associated with lipid-rich membranes; its anti-oxidative ability is highly effective in protecting membranes against lipid peroxidation, as peroxyl and alkoxyl radicals. The data obtained from the present study indicates that the tested extracts have an anti-lipid peroxidative character with similar reaction mechanisms to those of dl-α-tocopherol.

#### Cytotoxic activity [31]

As shown in table 4, human hepatocellular carcinoma (HEP-G2), colon adenocarcinoma (COLO 205) and gastric carcinoma (NCI-N87) were the most sensitive of all cell lines examined to the activities of the extracts, followed by the ovary adenocarcinoma (NIH:OVCAR-3). The aqueous extract of *Celtis occidentalis* L. leaves showed the strongest activity against HEP-G2 (ED_50_= 18.60 μg/mL), while the plant’s ethanolic extract was most active against COLO 205 and NCI-N87 (ED50= 24.80 and 15.80 μg/mL, respectively).

## Conclusions

This study provides strong evidence for the antioxidant activities of the leaves’ ethanolic extracts of *Celtis australis* L. and *Celtis occidentalis* L. that could be considered as two valuable medicinal plant species. The results obtained for the *n*-butanol fractions and the major isolated flavonoid, 2″-galactosylvitexin, indicated that the C-flavonoid glycosides in both studied plants are responsible for the scavenging effects through several mechanisms like hydrogen donating activity, inhibition of xanthine oxidase activity and superoxide anion production, as well as protection of membranes against lipid peroxidation. This could be potentially useful for the treatment of major free radical-induced degenerative diseases including brain dysfunction, inflammation, liver disorders and cancer. Further *in-vivo* studies could be done to support this point of view.

## Experimental

### Plant material

*Celtis australis* L. and *Celtis occidentalis* L. leaves were collected from El-Orman Botanical Garden and the Agricultural museum, Giza, Egypt. Dr. Mohamed El Gebaly, Taxonomist, National Research Center kindly verified identity of the plant material. Voucher specimens (G-02 and G-03) were kept in the Department of Pharmacognosy, Faculty of Pharmacy, Cairo University, Egypt.

### Chemicals

1,1-Diphenyl-2-picrylhydrazyl (DPPH), butylated hydroxytoluene (BHT), 2-thiobarbituric acid, ferrous sulphate, hydrogen peroxide, xanthine oxidase from bovine milk, allopurinol, nitroblue tetrazolium, 3-(4,5-dimethyl-1,3-thiazol-2-yl)-2,5-diphenyl-2*H*-tetrazol-3-ium bromide (MTT) and sodium 2-[4-(2-hydroxyethyl)piperazin-1-yl]ethanesulfonate (HEPES, for Kreb’s buffer) (Sigma, St. Louis, Mo, USA); coomassie plus protein assay reagent and albumin standard (Pierce, Rockford, IL, USA). dl-*α*-tocopherol (Nacalai, Tesuque, Tokyo, Japan); L-glutamine, 1% non-essential amino acids and 1% sodium pyruvate (Bio Whittaker, Walkersville, MD, USA). 10 % fetal bovine serum (FBS) (Gibco Br L, Rockville, MD, USA). TLC was performed on pre-coated sheets with silica gel F254 (Fluka, Sigma-Aldrich, Germany).Column chromatography was performed over silica gel H 60 for VLC (Sigma, St. Louis, Mo, USA.), sephadex LH-20 (Pharmacia Fine Chemicals, AB Uppsala, Sweden), polyamide and silica gel 60 (Fluka, Sigma-Aldrich, Germany). All other chemicals used were of analytical grade.

### Animals

Male Wistar rats (250–300 g) were handled according to international regulations. They were allowed to take standard laboratory diet and water ad libitum, and the animals were maintained at 24 °C with 12 h light period.

### Cell lines and culture media

Human hepatocellular carcinoma (HEP-G2), leukemia carcinoma (CCRF-CEM), colon adenocarcinoma (COLO 205), ovary adenocarcinoma (NIH:OVCAR-3) and gastric carcinoma (NCI-N87) cell lines were from the American Type Culture Collection (ATCC). Dulbecco’s Modified Eagle Medium (DMEM) (Gibco, Grand Island NY, USA). Eagle Minimum Essential Medium (EMEM) and Roswell Park Memorial Institute 1640 (RPMI) medium (Nissui Pharm. Co., Ltd., Tokyo, Japan); McCoy’s 5A modified medium (Sigma, St. Louis, Mo., USA).

### Preparation of extracts and fractions

The air-dried powdered leaves of *Celtis australis* L. (1.5 kg) and *Celtis occidentalis* L. (1.2 kg) were exhaustively extracted with 95% ethanol by percolation to give 225 g and 200 g dry residue, respectively. An aliquot of the concentrated extracts (150 g, each) was suspended in distilled water and partitioned successively with petroleum ether (32 and 34 g), CH_2_Cl_2_ (2 and 2.5 g), EtOAc (4 and 3.1 g), and *n*-BuOH (40 and 21 g).

For the aqueous extract, 50 g of the air-dried powdered leaves was boiled with distilled water (3 times, each for 10 min). The extracts were then lyophilized to give 12 and 10 g, respectively.

### Fractionation and separation of major components of Celtis australis L

The *n*-BuOH soluble fraction (20 g) was chromatographed on a silica gel H 60, VLC (Ø7 x 12.5 cm, 200 g) and eluted with CH_2_Cl_2_, gradients of CH_2_Cl_2_-EtOAc, EtOAc and gradients of EtOAc-MeOH up to pure MeOH. Fractions (200 mL, each) were collected and monitored by TLC. Similar fractions were pooled to give 6 collective fractions. Collective fraction 1 (2.1 g) was subjected to CC on polyamide using MeOH/H_2_O (20:80) v/v followed by CC on sephadex LH-20 using MeOH/(CH_3_)_2_CO/H_2_O (3:1:1) v /v/v to give compounds **1** (16 mg), **2** (7 mg) and **3** (60 mg). Collective fraction 2 (2 g) was purified by CC on silica gel 60 followed by CC on sephadex LH-20 using MeOH/(CH_3_)_2_CO/H_2_O (3:1:1) v/v/v to give compound **4** (20 mg). Collective fractions 3 (1.1 g) and 4 (4.2 g) were subjected to repeated CC on sephadex LH-20 using MeOH/H_2_O (50:50) v/v for elution to give compounds **5** (22 mg) and **6** (200 mg), respectively. Collective fraction 5 (750 mg) and 6 (1.5 g) were subjected to repeated CC on sephadex LH-20 using MeOH/(CH_3_)_2_CO/H_2_O (3:1:1) v/v/v to give compounds **7** (19 mg) and **8** (28 mg).

### Fractionation and separation of major components of Celtis occidentalis L

The *n*-BuOH soluble fraction (15 g) was chromatographed on a silica gel H 60, VLC (Ø7 x 12.5 cm, 190 g) and eluted with CH_2_Cl_2_, gradients of CH_2_Cl_2_-EtOAc, EtOAc and gradients of EtOAc-MeOH up to pure MeOH. Fractions (200 mL) were collected and monitored by TLC. Similar fractions were pooled to give 5 collective fractions. Collective fractions 1 (0.7 g) and 2 (2.84 g) were subjected to CC on polyamide using MeOH/H_2_O (20:80) v/v followed by CC on sephadex LH-20 using MeOH/(CH_3_)_2_CO/H_2_O (3:1:1) v/v/v to give compounds **1** (5 mg), **2** (21 mg) and **3** (80 mg). Collective fraction 3 (1.89 g) and 4 (1.56 g) were subjected to repeated CC on sephadex LH-20 using MeOH/H_2_O (50:50) v/v for elution followed by CC on sephadex LH-20 using MeOH/(CH_3_)_2_CO/H_2_O (3:1:1) v/v/v to give compounds **4** (16 mg) and **5** (8 mg), respectively. Collective fraction 5 (2.1 g) was subjected to repeated CC on sephadex LH-20 using MeOH (100%) for elution followed by CC on sephadex LH-20 using MeOH/(CH_3_)_2_CO/H_2_O (3:1:1) v/v/v to give compounds **6** (22 mg), **7** (32 mg) and **8** (11 mg).

#### 4‴-Rhamnosyl-2″-O-β-d-galactopyranosylvitexin (6-Deoxy-α-l-mannopyranosyl-(1→4)-β-d-galactopyranosyl-(1→2)-(1S)-1,5-anhydro- 1-[5,7-dihydroxy-2-(4-hydroxyphenyl)-4-oxo-4H-chromen-8-yl]-d-glucitol, **8**)

Yellow powder, mp: 289–291 °C. Rf: 0.21 (EtOAc/MeOH/H_2_O, 100:16:13 and 2 drops formic acid). UV/Vis λ_max_ (MeOH) nm: 272, 302sh, 330; (NaOMe): 280, 326, 393; (AlCl_3_): 277, 304, 350, 382; (AlCl_3_/HCl): 278, 303, 344, 384; (NaOAc): 280, 310sh., 336sh., 390; (NaOAc/H_3_BO_3_): 274, 308, 351, 400sh. HR-ESI/MS: *m/z* = 739.2085 (M – H) (calcd 739.2091), *m/z* = 741.2202 (M + H) (calcd 741.2210). ^1^H and ^13^C NMR: Table 1.

### Equipment

Electrothermal 9100 equipment was used for melting point determination. UV spectra were measured using a Shimadzu UV 1650 PC spectrophotometer. ESI-MS (negative and positive modes) were determined on Thermo Finnigan (ion trap) equipment. NMR spectra were recorded in DMSO-*d*_6_ with a Varian Mercury instrument (^1^HNMR, 300 MHz; ^13^CNMR, 75 MHz). Absorbance was measured on a multi well scanning spectrophotometer (Dynex MR 5000, Chantilly, VA, USA).

### In-vitro biological Evaluation

#### DPPH radical scavenging activity

The ability of the extracts to scavenge free radicals was determined according to the method of Hosny, *et al* 2002 [24]. In a 96-well plate, 10 μL of each sample or standard dissolved in ethanol (100 μg/mL) was added to 190 μL of 316 μM/mL DPPH solution. A blank was prepared using ethanol. After incubation at 30 °C for 30 min, the absorbance of each solution was measured at 517 nm. dl-α-tocopherol and BHT were used as positive controls. The scavenging activity of the samples was calculated as a percentage of free radical inhibition according to the formula:

$$\%\;\text{inhibition}=\frac{{\text{A}}_{\text{blank}}-{\text{A}}_{\text{sample}}}{{\text{A}}_{\text{blank}}}\times 100$$

where A_blank_ is the absorbance of the blank at zero time and A_sample_ is the absorbance of the sample after 30 min. All experiments were carried out in triplicate.

#### Xanthine oxidase-induced generation of superoxide radical

The effect of the tested samples was measured according to the method described by Luis Gongora *et al.*, 2003 [26] with slight modification. Superoxide was generated by oxidation of hypoxanthine (100 μM) with bovine milk xanthine oxidase in 1 mL of 10 mM KH_2_PO_4_-KOH buffer, pH 7.4, and was detected by the reduction of nitroblue tetrazolium (NBT) at 100 μM, followed spectrophotometrically at 560 nm. dl-α-tocopherol and BHT were used as positive controls. The effects of the test samples on enzyme activity was studied by measuring the uric acid formation from xanthine (2–25 μM) after 15 min incubation at 25 °C, while absorbance was measured at 295 nm using allopurinol as a reference standard. The inhibitory activity of the tested samples and isolated compound in terms of IC_50_, i.e. the concentration required for 50% inhibition of uric acid formation was calculated by linear regression analysis.

#### FeSO_4_/H_2_O_2_-stimulated lipid peroxidation in rat tissue homogenate [28, 29]

Male Wistar rats (250–300 g) were sacrificed, and the rat tissues (brain, heart and liver: 0.3–0.5 g) were rapidly removed and homogenized in 10 volumes of 15 mM Krebs buffer. Homogenates were centrifuged at 3000 x for 10 minutes at 4 °C to give supernatants containing (1.2 mg of protein/ ml; brain), (1.7 mg of protein/ ml; heart) and (2.5 mg of protein/ ml; liver) using Coomassie plus protein assay reagent and albumin standard as determined by the Bradford method [32]. During aerobic incubation of the tissue homogenates, MDA released reacts with thiobarbituric acid (TBA) to give a pink colour. The capability of the samples to inhibit MDA formation is used as a measure of their antioxidant activity. The pink colour complex of thiobarbituric acid reacting substance (TBARS) is measured at 532 nm for the test samples and positive standards (dl-α-tocopherol and BHT) (200 μg/mL), as well as, 2″-*O*-*β*-galactopyranosylvitexin (100 μg/mL). The results were expressed as nanomoles of MDA equivalents per milligram of protein of rat (brain, heart and liver) homogenates. All measurements were done in triplicate. The capability to inhibit MDA formation was calculated using the following equation:

$$\text{Inhibition effects }(\%)=1-\frac{\text{MDA in tissue homogenate with}\;\text{test extracts}}{\text{MDA in tissue homogenate without test extracts}}\times 100$$

#### Cytotoxic activity

Cell lines, HEP-G2, were cultured in DMEM containing 10% heat-inactivated FBS. CCRF-CEM and NCI-N87 cells were cultured in RPMI1640 medium supplemented with 1% L-glutamine, 1% non-essential amino acids, 1% sodium pyruvate and 10% FBS. The COLO 205 human colon carcinoma cells were grown in McCoy’s 5A modified medium supplemented with 1% L-glutamine. NIH:OVCAR-3 cells were cultured in EMEM containing Earle’s salts heated and sublimated with amino acids and 10% FBS. All cell lines were cultured in T75 Falcon flasks in a humidified atmosphere with 5% CO_2_, at 37°C. Cellular viability was determined using the standard MTT colorimetric assay [31]. The assay is based on reduction of MTT by the mitochondrial dehydrogenase of viable cells to give a blue formazan product that can be measured spectrophotometrically at 550 nm. The 50 % effective dose (ED_50_) is calculated.

### Statistical Analysis

All data were expressed as mean ± SE. Student’s t-test [33] was applied for detecting the significance of difference between each sample; P < 0.05 was taken as the level of significance.

## Figures and Tables

**Fig. 1:**
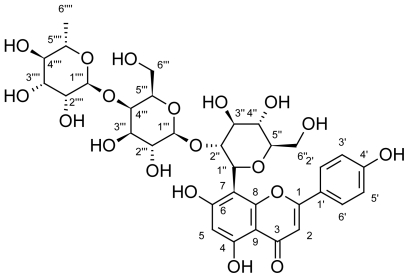
Structure and atom numbering of compound **8**.

**Tab. 1:** ^1^H and ^13^C NMR data of compound **8** (DMSO-*d*_6_, 300, 75 MHz)

No.	Compound 8		No.	Compound 8	
	δH^a^			δH^a^	No.
2		163.7	3″		75.8
3	6.70, s	102.5	4″		70.8
4		182.0	5″		81.5
5		160.1	6″		61.1
6	6.21, s	97.9	2″-*O*-Gal 1‴	3.99, d (9)	101.4
7		162.4	2‴		72.3
8		104.3	3‴		74.0
9		156.3	4‴		67.2
10		103.9	5‴		75.0
1′		121.8	6‴		58.1
2′	8.01, d (8.7)	128.8	4‴-*O*-Rha 1″″	4.90, s	100.2
3′	6.89, d (9)	115.8	2″″		70.4
4′		160.9	3″″		70.6
5′	6.89, d (9)	115.8	4″″		73.2
6′	8.01, d (8.7)	128.8	5″″		68.1
8-*C*-Glc 1″	4.76, d (9.9)	71.7	6″″	1.19, d (6)	17.5
2″		78.9			

a Coupling constants (J in Hertz) are in parentheses.

**Tab. 2:** Effects of the tested samples on the in vitro free radical generation

Bioassay	DPPH % inhibition	Xanthine oxidase^a^ IC_50_ (μM)
Ethanolic extract (CA)	67.2 ± 2.10	76.8 ± 2.90
Ethanolic extract (CO)	58.5 ± 1.50	92.1 ± 3.55
Aqueous extract (CA)	55.6 ± 2.10	70.2 ± 2.18
Aqueous extract (CO)	48.5 ± 1.35	95.5 ± 3.70
*n-*butanol fraction (CA)	70.3 ± 2.20	27.2 ± 2.10
*n-*butanol fraction (CO)	65.9 ± 1.96	38.5 ± 1.78
2″-*O*-β-galactopyranosylvitexin	84.8 ± 2.44	24.2 ± 1.95
dl*-*α-tocopherol	66.5 ± 2.75	78.5 + 2.88
BHT	55.3 ± 1.50	130.7 + 4.35
Allopurinol	–	18.0 ± 0.25

a Uric acid production for controls was (61.0 ± 1.9 nmol/min).

All tested samples and positive controls were tested at 100 μg/mL.

Values are presented as mean ± SE of 3-test sample observation. P < 0.05 for all values.

CA = Celtis australis L.; CO = Celtis occidentalis L.

**Tab. 3:** Inhibition effects of the tested samples on FeSO_4_/H_2_O_2_-stimulated lipid peroxidation (MDA production) in rat tissue homogenates *in vitro*

Bioassay	Inhibition (%)^a^		
Sample	Brain	Heart	Liver
Ethanolic extract (CA)^b^	80.85 ± 1.15	55.30 ± 1.80	68.30 ± 1.83
Ethanolic extract (CO)^b^	75.60 ± 1.97	50.22 ± 1.80	70.90 ± 2.15
Aqueous extract (CA)^b^	55.10 ± 2.28	52.40 ± 2.18	43.90 ± 2.25
Aqueous extract (CO)^b^	43.50 ± 1.55	48.75 ± 1.28	32.75 ± 1.80
*n*-butanol fraction (CA)^b^	66.18 ± 2.10	54.10 ± 1.80	49.75 ± 1.76
*n*-butanol fraction (CO)^b^	64.82 ± 2.05	60.05 ± 1.85	53.10 ± 1.80
2″-*O*-β-galactopyranosylvitexin^c^	80.78 ± 3.12	71.50 ± 2.35	78.25 ± 3.10
dl-α-tocopherol^b^	62.90 ± 2.18	58.70 ± 1.80	71.10 ± 2.25
BHT^b^	52.48 ± 1.72	43.23 ± 1.65	53.20 ± 1.65

a Values are presented as mean ± SE of 3-test sample observations, P < 0.05 for all values;

b 200 μg/mL;

c 100 μg/mL; CA = *Celtis australis* L.; CO = *Celtis occidentalis* L.

**Tab. 4:** ED_50_ (μg/mL) of the tested samples on the selected cell lines^a^

Sample	HEP-G2	CCRF-CEM	COLO 205	NCI-N87	NIH-OVAR-3
Ethanolic extract (CA)	26.10 ± 0.20	> 100	25.65 ± 0.20	45.15 ± 0.25	77.65 ± 0.52
Ethanolic extract (CO)	39.85 ± 0.25	> 100	24.80 ± 0.20	15.80 ± 0.05	68.50 ± 0.45
Aqueous extract (CA)	26.90 ± 0.20	77.50 ± 0.53	63.45 ± 0.40	35.10 ± 0.15	72.77 ± 0.48
Aqueous extract (CO)	18.60 ± 0.08	79.54 ± 0.47	26.60 ± 0.25	23.55 ± 0.10	76.10 ± 0.55

a Values are presented as mean ± SE of 2 test sample observation, compared with that of control group (p < 0.05) for all values; HEP-G2…Human hepatocellular carcinoma; CCRF-CEM…leukemia carcinoma; COLO 205…colon adenocarcinoma; NIH-OVCAR-3…ovary adenocarcinoma; NCI-N87…gastric carcinoma; CA…Celtis australis L.; CO…Celtis occidentalis L.
